# Effects of Web-Based Mindfulness-Based Interventions on Anxiety, Depression, and Stress Among Frontline Health Care Workers During the COVID-19 Pandemic: Systematic Review and Meta-Analysis

**DOI:** 10.2196/44000

**Published:** 2023-08-29

**Authors:** Jia-ming Yang, Hua Ye, Yi Long, Qiang Zhu, Hui Huang, Yan-biao Zhong, Yun Luo, Lei Yang, Mao-yuan Wang

**Affiliations:** 1 Department of Rehabilitation Medicine First Affiliated Hospital of Gannan Medical University Ganzhou China; 2 Gannan Medical University Ganzhou China; 3 Ganzhou Intelligent Rehabilitation Technology Innovation Center Ganzhou China; 4 Ganzhou Key Laboratory of Rehabilitation Medicine Ganzhou China; 5 Department of Rehabilitation Medicine The Second People’s Hospital of Kunming Kunming China; 6 Department of Geriatric Medicine The Second People’s Hospital of Kunming Kunming China; 7 Kunming Institute of Geriatrics The Second People’s Hospital of Kunming Kunming China

**Keywords:** web-based, mindfulness-based intervention, COVID-19, health care worker, mental disorder

## Abstract

**Background:**

Since 2019, the COVID-19 outbreak has spread around the world, and health care workers, as frontline workers, have faced tremendous psychological stress.

**Objective:**

The purpose of this study is to explore whether web-based mindfulness-based interventions continue to have a positive impact on anxiety, depression, and stress among health care workers during the COVID-19 pandemic.

**Methods:**

The inclusion criteria were as follows: (1) participants were frontline health care workers during the COVID-19 pandemic; (2) the experimental group was a web-based mindfulness-based intervention; (3) the control group used either general psychological intervention or no intervention; (4) outcome indicators included scales to assess anxiety, depression, and stress; and (5) the study type was a randomized controlled study. Studies that did not meet the above requirements were excluded. We searched 9 databases, including Web of Science, Embase, PubMed, Cochrane Library, Scopus, ScienceDirect, SinoMed, China National Knowledge Infrastructure (CNKI), and Wanfang Database, for randomized controlled studies on the effects of web-based mindfulness-based interventions on common mental disorder symptoms among health care workers from January 1, 2020, to October 20, 2022. The methodological quality of the included studies was assessed using the Physiotherapy Evidence Database scale. The Cochrane risk of bias tool was used to assess the risk of bias. Subgroup analysis was used to look for sources of heterogeneity and to explore whether the results were the same for subgroups under different conditions. Sensitivity analysis was used to verify the stability of the pooled results.

**Results:**

A total of 10 randomized controlled studies with 1311 participants were included. The results showed that web-based mindfulness-based interventions were effective in reducing the symptoms of anxiety (standard mean difference [SMD]=–0.63, 95% CI –0.96 to –0.31, *P*<.001, *I*^2^=87%), depression (SMD=–0.52, 95% CI –0.77 to –0.26, *P*<.001, *I*^2^=75%), and stress (SMD=–0.20, 95% CI –0.35 to –0.05, *P*=.01, *I*^2^=58%) among health care workers during the COVID-19 pandemic, but with wide CIs and high heterogeneity.

**Conclusions:**

Web-based mindfulness-based interventions may be effective in reducing the symptoms of anxiety, depression, and stress among frontline health care workers during the COVID-19 pandemic. However, this effect is relatively mild and needs to be further explored by better studies in the future.

**Trial Registration:**

PROSPERO CRD42022343727; https://www.crd.york.ac.uk/prospero/display_record.php?RecordID=343727

## Introduction

The COVID-19 outbreak first occurred in China in late 2019, followed by a succession of cases reported worldwide. COVID-19 is characterized by rapid onset, infectiousness, and rapid disease progression, posing a serious threat to human life and health [1]. To save the lives of patients with COVID-19 and stop the spread of the disease, health care workers are engaged in the fight against the pandemic. However, prolonged contact with patients affected by COVID-19 and working on COVID-19–related matters exposes health care workers to the virus, making them a susceptible population. Unfortunately, many health care workers have contracted COVID-19 and have even lost their lives in the fight against the pandemic [2,3]. The heavy workload and exposure to the risk of infection place health care workers under great psychological stress [4]. Consequently, health care workers are prone to mental disorders such as posttraumatic stress, anxiety, depression, and stress [5], which affect their daily diet and sleep and manifest in loss of appetite and insomnia [6,7], thus causing damage to their physical health. It is reported that the prevalence of anxiety among frontline health care workers during the COVID-19 pandemic was reported to be 25.8%, the prevalence of depression was 24.3%, and the prevalence of stress was 45% [8]. It is evident that during the COVID-19 pandemic, most frontline health care workers were affected by mental disorders. Therefore, in the fight against the COVID-19 pandemic, it is crucial to mitigate the symptoms of common mental disorders among frontline health care workers through some strategies to safeguard their physical and psychological health.

There are many intervention strategies for the management of common mental disorders among health care professionals, such as yoga [9], mindfulness-based intervention [10], auricular acupuncture [11], and guided imagery [12]. Among them, mindfulness-based interventions have gained enough attention and are believed to be effective in reducing the symptoms of common mental disorders, such as anxiety, depression, and stress [13,14]. Currently, a lot of interventions have been derived based on mindfulness therapy, such as mindfulness-based stress reduction (MBSR) and mindfulness-based cognitive therapy (MBCT) [15]. They not only reduced the symptoms of common mental disorders in participants but also improved their job satisfaction and sleep quality [14,16]. Generally, mindfulness-based interventions require face-to-face interaction between the interventionist and participant [17]. During the COVID-19 pandemic, however, it was evident that a face-to-face approach would not work. Consequently, attention has gradually shifted to web-based mindfulness-based interventions [18-20]. The effects of web-based mindfulness-based interventions on the symptoms of common mental disorders have been studied by many scholars before 2020 [14,21,22]. A systematic review and meta-analysis conducted in 2016 showed that web-based mindfulness-based interventions have beneficial effects on reducing the symptoms of common mental disorders, particularly stress [23]. However, the results of a systematic review of medical students revealed insufficient evidence to suggest that web-based mindfulness-based interventions can be used to treat depression and anxiety [24]. Therefore, there is a lack of uniform conclusions regarding the efficacy of web-based mindfulness-based interventions on the symptoms of common mental disorders. Given the complex trend of the COVID-19 pandemic, health care workers are often under more psychological stress than the general population because they are on the frontlines of the fight against the pandemic. Therefore, is the web-based mindfulness-based intervention effective in reducing symptoms of common mental disorders such as anxiety, depression, and stress among frontline health care workers during the COVID-19 pandemic? This study aimed to systematically review previously published articles to explore whether web-based mindfulness-based interventions could reduce the symptoms of anxiety, depression, and stress among frontline health care workers during the COVID-19 pandemic.

## Methods

According to the PRISMA (Preferred Reporting Items for Systematic Reviews and Meta-Analyses) guidelines [25,26], this study has been registered in PROSPERO (CRD42022343727). On October 20, 2022, we updated the search date and added retrieval of the ScienceDirect database.

### Search Strategies

We systematically searched published articles on the Web of Science, Embase, PubMed, Cochrane Library, Scopus, ScienceDirect, SinoMed, China National Knowledge Infrastructure (CNKI), and Wanfang Database without language restrictions from January 1, 2020, to October 20, 2022. Based on the Medical Subject Headings, we used subject terms and free-text terms for the literature search. The subject terms used were mindfulness, COVID-19, health care worker, anxiety, depression, and stress. The search strategies for each database are shown in Multimedia Appendix 1. Furthermore, to prevent the omission of relevant literature, we screened the references of the included studies to find clinical studies that met the inclusion criteria.

### Study Selection

The relevant studies were independently searched by 2 authors (QZ and HH). If there were any disputes, the help of a third author (J-mY) was sought. Based on the PICOS (population, intervention, comparison, outcomes, and study design) principles [27], we established the following inclusion criteria for the study:

1. Population: frontline health care workers (eg, doctors, nurses, and other medical workers) during the COVID-19 pandemic.
2. Intervention: web-based mindfulness-based intervention, including MBSR, MBCT, and mindfulness meditation.
3. Comparison: the control group used either general psychological intervention or no intervention.
4. Outcomes: outcome indicators include scales to assess anxiety, depression, and stress, such as the Self-Rating Anxiety Scale (SAS), the Self-Rating Depression Scale (SDS), and so on.
5. Study design: only randomized controlled studies were included.

Studies that did not meet the above requirements were excluded.

### Data Extraction

Two authors (HY and Y Long) independently extracted the data from the included studies into an Excel (Microsoft Corp) spreadsheet and then summarized the data in a table. If there were any inconsistencies, a third author (J-mY) was involved, and a unified opinion was formed.

The data extracted included (1) first author and year of publication; (2) country of participants; (3) occupational roles of participants; (4) groups and sample size, sex, and age of participants; (5) outcome measurement time points; (6) duration of intervention; (7) main outcomes; and (8) outcome assessment scale.

### Quality Assessment

Two authors (HY and Y Long) independently completed the methodological quality assessment of the included studies using the Physiotherapy Evidence Database (PEDro) scale. If they did not agree to the scoring of the same study, a session was held to resolve the issue. If the problem remained unresolved, a third author (J-mY) reached a consensus. There were eleven items on the PEDro scale: (1) eligibility criteria and source, (2) random allocation, (3) concealed allocation, (4) baseline comparability, (5) participant blinding, (6) therapist blinding, (7) assessor blinding, (8) adequate follow-up (>85%), (9) intention-to-treat analysis, (10) between-group statistical comparisons, and (11) point and variability measurements. It is important to note that the scores on the PEDro scale are cumulative from items 2 to 11, excluding item 1, with a minimum score of 0 and a maximum score of 10. Based on the scores of each study, we classified study quality into four levels: (1) poor, scores less than 4; (2) fair, scores between 4 and 5; (3) good, scores between 6 and 8; and (4) excellent, scores between 9 and 10 [28].

### Risk of Bias

Two authors (Y-bZ and Y Luo) independently assessed the risk of bias in the included studies using the Cochrane risk of bias tool [29]. Any disagreements were resolved at the end of the discussion. There are seven items in the bias risk table: (1) random sequence generation (selection bias), (2) allocation concealment (selection bias), (3) blinding of participants and personnel (performance bias), (4) blinding of outcome assessment (detection bias), (5) incomplete outcome data (attrition bias), (6) selective reporting (reporting bias), and (7) other bias. Each item was classified as “low risk,” “high risk,” or “unclear risk.” Furthermore, if a sufficient number (n≥7) of studies were included, a funnel plot analysis was performed to assess publication bias.

### Grading of Evidence Level

Based on the GRADE (Grading of Recommendations Assessment Development and Evaluation) criteria [30], 2 authors (J-mY and HY) assessed the level of evidence for the main outcome. The criteria are applicable to randomized trials and observational trials. For randomized trials, the initial quality grade for the outcome was high. The level of evidence was then appropriately downgraded based on the assessment of 5 dimensions, namely risk of bias, inconsistency, imprecision, indirectness, and publication bias. According to the results of the assessment, the level of evidence was categorized into 4 grades: “high,” “moderate,” “low,” and “very low.”

### Data Synthesis and Statistical Analyses

Review Manager (version 5.3; The Cochrane Collaboration) was used to perform the statistical analysis. In this study, we extracted the postintervention data for statistical analysis because the baseline differences between studies were not significant. For continuous data, we entered the pre-extracted mean, SD, and sample size into statistical software. For the same outcome, if the method of assessing the outcome was the same across studies, the mean difference was used to estimate the effect size and the 95% CIs to indicate the CI range; if not, the standard mean difference (SMD) was used. In all analyses, the magnitude of heterogeneity was analyzed according to the *I*^2^ statistic, with *I*^2^<50% indicating low heterogeneity and *I*^2^≥50% indicating high heterogeneity. If the *P* value of the heterogeneity test was less than .05, the random-effects model was used; if the *P* value was greater than or equal to .05, the fixed-effects model was used [31]. In addition, subgroup analysis was used to look for sources of heterogeneity and to explore whether the results were the same for subgroups under different conditions; sensitivity analysis was performed to verify the stability of the results.

## Results

### Study Selection

**Figure 1:**
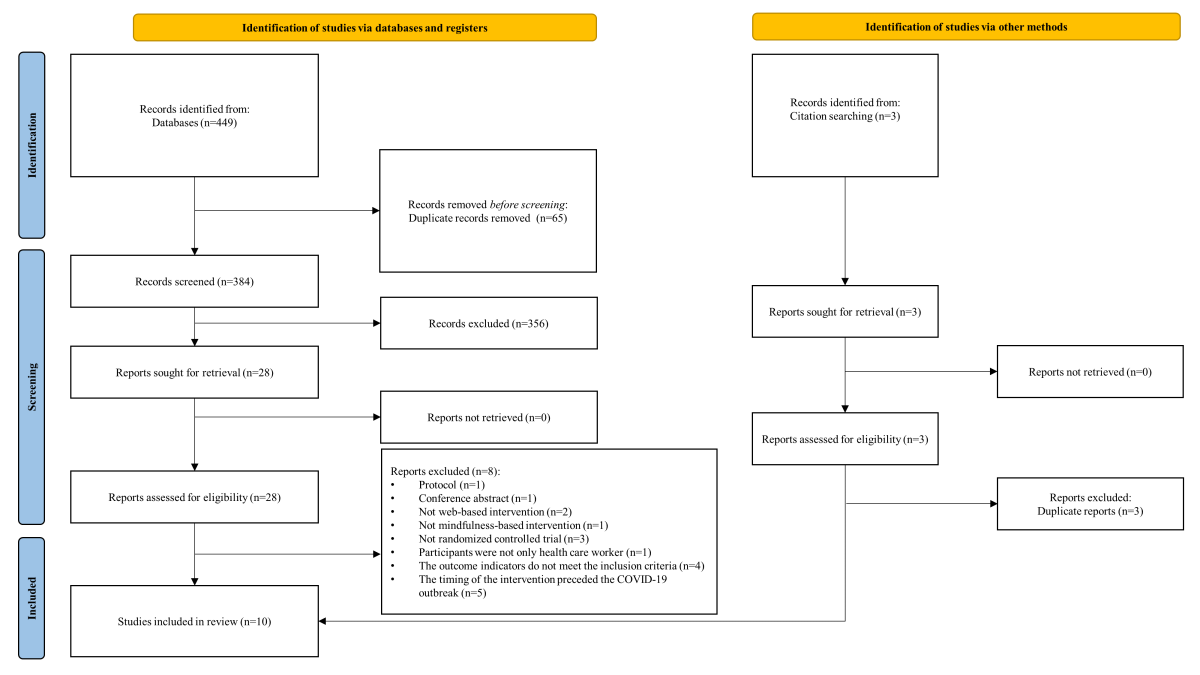
Flowchart of literature screening.

After a systematic search of 8 databases, we retrieved 449 articles, including 13 in PubMed, 46 in Web of Science, 3 in Embase, 124 in the Cochrane Library, 19 in Scopus, 206 in ScienceDirect, 19 in SinoMed, 13 in CNKI, and 6 in the Wanfang Database. In addition, 3 papers came from the citation search. First of all, after eliminating duplicate articles by EndNote software (Thomson Corp), 387 articles remained. Next, we read the titles and abstracts, and 359 articles were removed, with 28 remaining. We then read the full text, and 18 articles were excluded, leaving 10 articles. All literature selection processes were done independently by 2 authors using EndNote software. The list of excluded studies and the reasons for their exclusion are shown in Multimedia Appendix 2. As a result, 10 randomized controlled studies were ultimately included in the study [32-41] (Figure 1).

### Study Characteristics

Of the 10 articles, 5 were in English [32-36] and 5 were in Chinese [37-41]. For convenience, all the Chinese references have been translated into English. Three studies recruited only nurses [34,37,41], and the remaining studies recruited 2 or more occupations. The total duration of the intervention was 8 weeks in 3 studies [36,38,39], 4 weeks in 3 studies [37,40,41], 16 days in 1 study [35], and 2 weeks in 1 study [32]. The duration of the intervention was the number of sessions in 2 studies: 7 sessions in 1 study [33] and only 1 session in another study [34]. Three studies assessed anxiety and depression used the SAS and SDS, respectively [37,40,41]; 3 studies assessed anxiety and depression used the 7-item Generalized Anxiety Disorder Scale and Patient Health Questionnaire-9, respectively [35,38,39]; 1 study assessed depression, anxiety and stress used the Depression, Anxiety, and Stress Scale-21 [32]; 1 study used the Hospital Anxiety and Depression Scale (HADS) to assess depression, and the HADS and the Short Health Anxiety Inventory (SHAI) to assess anxiety, respectively [33]; 1 study assessed depression and anxiety used the Goldberg Depression Scale and Goldberg Anxiety Scale, respectively [36]; 1 study assessed stress used the State Anxiety Inventory-I [34]; and 1 study assessed stress used the Perceived Stress Scale [35] (Table 1).

**Table 1:** Baseline characteristics of randomized controlled studies included in this study.

Study	Country	Occupational role	Groups (sample), age: mean (SD)	Time points of assessment	Duration of intervention	Outcomes	Scale
Fiol-DeRoque et al [32]	Spain	Physicians, nurses, nurse assistants, and other	EG^a^: PsyCovidApp intervention (n=221), age: 42.07 (SD 11.0) years; CG^b^: Control App (n=215), age: 40.62 (SD 9.6) years	Baseline, Week 2	2 weeks	Depression, anxiety, and stress	DASS-21^c^
Ghazanfarpour et al [33]	Iran	All health care workers	EG: Standard cognitive-behavioral and mindfulness-based techniques (n=51), age: —^d^; CG: No intervention (n=44), age: —	Pretest, Posttest	7 sessions	ARC^e,f^, ALI^f,g^, DRC^h^, ANC^i^	HADS^j^, SHAI^k^
Li et al 2020 [35]	China	Doctors, nurses, and others	EG: Brief mindfulness meditation (n=87); age: 21-60 years; CG: No intervention (n=47); age: 21-60 years	Pretest, Posttest	16 days	Anxiety, stress, and depression	PHQ-9^l^, GAD-7^m^, PSS^n^
Santamaría-Peláez et al [36]	Spain	Physicians or medicines, and nurses	EG1: 4-weekly MBSR^o^ (n=24); age: 47.66 (SD 13.67) years; EG2: 8-weekly MBSR (n=37); age: 35.73 (SD 12.04) years; CG: No intervention (n=51); age: 40.34 (SD 13.22) years	Pretest, Posttest, 3 months after intervention	4 weeks and 8 weeks	Anxiety, depression	GAS^p^, GDS^q^
Yıldırım et al [34]	Turkey	Nurses	EG: Mindfulness-based breathing and music therapy for 30 minutes (n=52), age: 27.55 (SD 5.24) years; CG: Relaxing in a quiet and calm setting for 30 minutes (n=52), age: 29.11 (SD 6.57) years	Pretest, Posttest	Single session	Stress^f^	STAI-I^r^
Zeng et al [40]	China	Doctors and nurses	EG: MBSR (n=40), age: 36.25 (SD 5.01) years; CG: WeChat-based psychological intervention (n=40), age: 35.28 (SD 4.66) years	Baseline, Week 4	4 weeks	Depression^f^, anxiety^f^	SAS^s^, SDS^t^
Huang et al [39]	China	Doctors, nurses, and others	EG: MBSR (n=58), age: 31.74 (SD 5.41) years; CG: Psychological health education (n=60), age: 32.78 (SD 7.23) years	Baseline, Week 8	8 weeks	Depression^f^, anxiety^f^	PHQ-9, GAD-7
Tong et al [41]	China	Nurses	EG: MBSR and general psychological intervention (n=16), age: 25.25 (SD 5.13) years; CG: General psychological intervention (n=16), age: 24.94 (SD 4.77) years	Baseline, Week 4	4 weeks	Depression^f^, anxiety^f^	SAS, SDS
Wang and Chen [37]	China	Nurses	EG: MBSR and general psychological intervention (n=50); CG: general psychological intervention (n=50); total age: 28.99 (SD 8.63) years	Baseline, Week 4	4 weeks	Depression^f^, anxiety^f^	SAS, SDS
Zou et al [38]	China	All health care workers	EG: mindfulness meditation combined with positive psychological intervention (n=50), age: 46.84 (SD 5.26) years; CG: not receive any treatment (n=50), age: 46.24 (SD 4.83) years	Baseline, Week 8	8 weeks	Depression^f^, anxiety^f^	PHQ-9, GAD-7

^a^EG: experimental group.

^b^CG: control group.

^c^DASS-21: Depression, Anxiety, and Stress Scale-21.

^d^Not available.

^e^ARC: anxiety related to coronavirus.

^f^Compared with the control group, outcome indicators decreased after the intervention (*P*<.05).

^g^ALI: anxiety of likelihood of illness.

^h^DRC: depression related to coronavirus.

^i^ANC: anxiety of negative consequences.

^j^HADS: the Hospital Anxiety and Depression Scale.

^k^SHAI: Short Health Anxiety Inventory.

^l^PHQ-9: Patient Health Questionnaire-9.

^m^GAD-7: 7-item Generalized Anxiety Disorder Scale.

^n^PSS: Perceived Stress Scale.

^o^MBSR: mindfulness-based stress reduction.

^p^GAS: Goldberg Anxiety Scale.

^q^GDS: Goldberg Depression Scale.

^r^STAI-I: State Anxiety Inventory-I.

^s^SAS: Self-Rating Anxiety Scale.

^t^SDS: Self-Rating Depression Scale.

The financial support and disclosures of interest in the included studies are shown below. Six studies indicated that they were funded [32,33,35,36,39,40], and 5 claimed no conflict of interest between the authors [32-36]. The remaining studies did not mention financial support or conflicts of interest.

### Web-Based Mindfulness-Based Intervention Protocol

One study asked participants to use PsyCovidApp (a self-managed and self-guided psychoeducational mobile-based intervention with no therapist support) for a self-management psychological intervention based on cognitive behavioral therapy and mindfulness therapy [32]. One study divided the participants in the experimental group into 21 WhatsApp groups for 7 intervention sessions. The content of the 7 sessions was selected according to standard cognitive-behavioral and mindfulness techniques. The duration of each session varied from 45 to 90 minutes of communication [33]. One study divided the intervention group into 9 subgroups, each of which underwent a single session of mindfulness-based breathing and music therapy for approximately 30 minutes [34]. One study asked participants to practice 15 minutes of brief mindfulness meditation daily for 16 days [35]. The experimental group in 3 studies used MBSR training [36,39,40], 2 studies used MBSR combined with general psychological intervention [37,41], and 1 study used mindfulness meditation combined with positive psychological intervention [38]. Details of the interventions for the experimental and control groups of each study are shown in Multimedia Appendix 3.

### Quality of the Included Studies

The PEDro scores and score details of each study are presented in Table 2. The PEDro scores for the 10 studies were as follows: 1 study scored 4 (quality grade: fair) [35], 1 study scored 5 (quality grade: fair) [36], 6 studies scored 6 (quality grade: good) [34,37-41], 1 study scored 7 (quality grade: good) [33], and 1 study scored 8 (quality grade: good) [32]. All studies had a random allocation, baseline comparisons, between-group comparisons, and point and variability measures. Two studies referred to the random assignment of participants by individuals who did not participate in the study (concealed allocation) [32,33]. Blinding was not mentioned in any of the remaining studies, except for one study that used double-blinding [32] and 3 studies that used single-blinding [33,34,36]. Two studies did not have adequate follow-up numbers (<85%) [35,36]. All participants in 5 studies completed the experiments according to the established study protocol [37-41].

**Table 2:** The Physiotherapy Evidence Database (PEDro) score of each study.

Study	(1)^a^	(2)	(3)	(4)	(5)	(6)	(7)	(8)	(9)	(10)	(11)	Score	Study quality
Fiol-DeRoque et al [32]	Yes^b^	1	1	1	1	0	1	1	0	1	1	8	Good
Ghazanfarpour et al [33]	Yes	1	1	1	0	0	1	1	0	1	1	7	Good
Li et al 2020 [35]	Yes	1	0	1	0	0	0	0	0	1	1	4	Fair
Santamaría-Peláez et al [36]	Yes	1	0	1	0	0	1	0	0	1	1	5	Fair
Yıldırım et al [34]	Yes	1	0	1	0	0	1	1	0	1	1	6	Good
Zeng et al [40]	Yes	1	0	1	0	0	0	1	1	1	1	6	Good
Huang et al [39]	Yes	1	0	1	0	0	0	1	1	1	1	6	Good
Tong et al [41]	Yes	1	0	1	0	0	0	1	1	1	1	6	Good
Wang and Chen [37]	Yes	1	0	1	0	0	0	1	1	1	1	6	Good
Zou et al [38]	Yes	1	0	1	0	0	0	1	1	1	1	6	Good

^a^(1) Eligibility criteria were specified, (2) random allocation, (3) concealed allocation, (4) baseline comparability, (5) participant blinding, (6) therapist blinding, (7) assessor blinding, (8) adequate follow-up (>85%), (9) intention-to-treat analysis, (10) between-group statistical comparisons, and (11) point and variability measurements.

^b^Yes, one point; No, score 0. A total PEDro score is achieved by adding the ratings of (2) to (11) for a combined total score between 0 and 10.

### Risk of Bias in Included Studies

**Figure 2:**
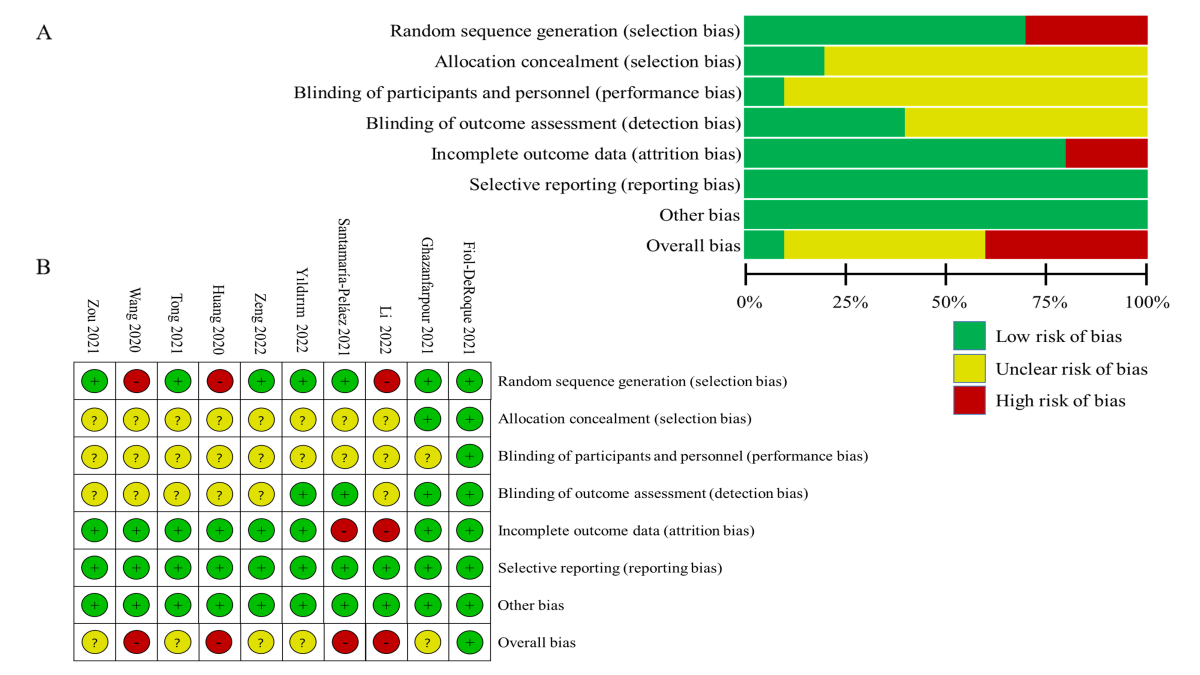
Risk of bias graph and summary of included studies. (A) Risk of bias graph shows the overall risk of bias in each domain. (B) Risk of bias summary indicates the risk of bias in each domain for each study.

Figure 2 [32-41] shows the risk of bias results of the 10 studies based on the Cochrane risk of bias tool. Three studies did not mention the specific method of random sequence generation (high risk of bias) [35,37,39]; 4 studies blinded the assessors [32-34,36] and only 1 study stated that it blinded the participants (low risk of bias) [32]; 2 studies used allocation concealment (low risk of bias) [32,33]; and outcome data of 2 studies were incomplete (high risk of bias) [35,36]. No studies had selection bias or other bias (low risk of bias). As a result, the overall bias was low for 1 study [32], high for 4 studies [35-37,39], and unclear for other studies.

### Effects of a Web-Based Mindfulness-Based Intervention on Outcomes

Of the 10 studies, 9 investigated the effect of web-based mindfulness-based on anxiety and depression [32,33,35-41], and 3 measured the effect on stress [32,34,35]. Among them, the means and SDs of data from 2 studies could not be extracted [38,39], so for anxiety and depression, only data from 7 studies were pooled. The data were meta-analyzed using the SMD because the assessment scales used varied across studies. For anxiety and depression, we used a random-effects model to estimate effect values because the *P* value for heterogeneity was less than .05; for stress, we used a fixed-effects model because the *P* value was greater than .05.

**Figure 3:**
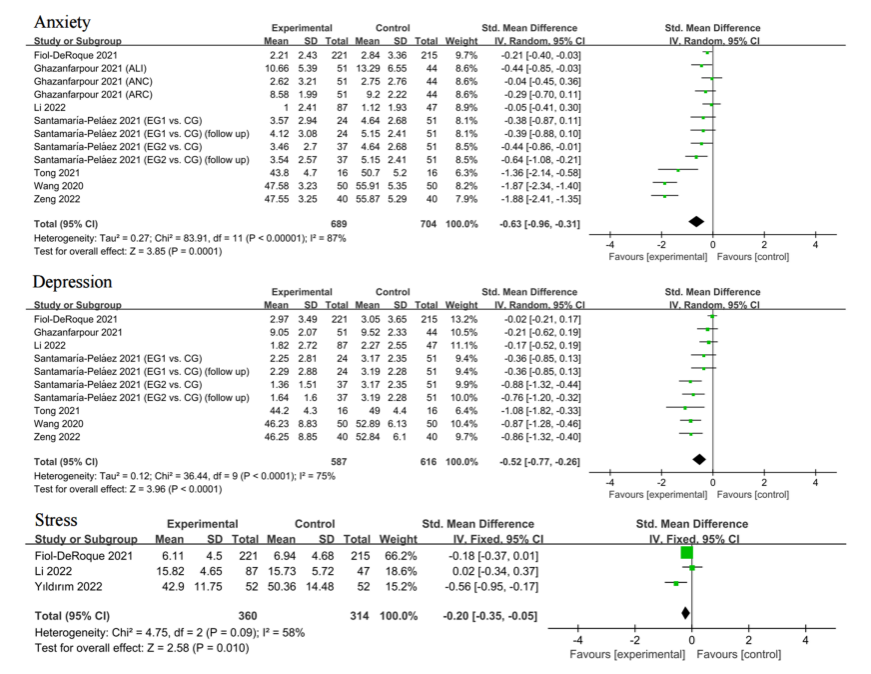
Forest plots of the effects of web-based mindfulness-based interventions on anxiety, depression, and stress [33-38,41,42].

The results of forest plots showed that the web-based mindfulness-based interventions significantly reduced anxiety (SMD=–0.63, 95% CI –0.96 to –0.31, *P*<.001, *I*^2^=87%), depression (SMD=–0.52, 95% CI –0.77 to –0.26, *P*<.001, *I*^2^=75%), and stress (SMD=–0.20, 95% CI –0.35 to –0.05, *P*=.01, *I*^2^=58%) among frontline health care workers during the COVID-19 pandemic (Figure 3). Furthermore, although we did not combine data from the studies of Zou et al [38] and Huang et al [39], their results both suggest that web-based mindfulness-based interventions reduced anxiety and depression in health care workers.

### Subgroup Analysis

First, we performed subgroup analyses for anxiety and depression according to the duration of the intervention. The duration of intervention for the 2 subgroups was 7 to 16 days and 4 to 8 weeks, respectively. For anxiety, both subgroups showed that the web-based mindfulness-based interventions significantly reduced the anxiety levels of health care workers (*P*<.05). For depression, mindfulness-based interventions lasting 4 to 8 weeks significantly reduced depression levels (SMD=–0.73, 95% CI –0.91 to –0.54, *P*<.001, *I*^2^=6%), but interventions lasting 7 to 16 days did not (SMD=–0.08, 95% CI –0.23 to 0.08, *P*=.33, *I*^2^=0%). It is important to note that for depression, the heterogeneity was 0% and 6% for the 2 subgroups, suggesting that the heterogeneity may come from the duration of intervention. (Multimedia Appendix 4)

Second, we performed subgroup analyses for anxiety and depression according to the types of scales. The 2 subgroups were SAS or SDS and other scales, respectively. The results showed that both subgroups reduced the anxiety and depression levels of health care workers (*P*<.05). It is worth noting that for anxiety, the heterogeneity was 0% for both subgroups, suggesting that the heterogeneity perhaps mainly comes from the types of scales. (Multimedia Appendix 5)

### Sensitivity Analysis

By removing the included studies one by one, we performed sensitivity analyses for anxiety, depression, and stress. The results showed that the effects of web-based mindfulness-based interventions on anxiety and depression were very stable, with *P* values consistently less than .05. However, for stress, the result became meaningless (*P*>.05) when the study by Fiol-DeRoque et al [32] or Yıldırım et al [34] was removed.

### Publication Bias

**Figure 4:**
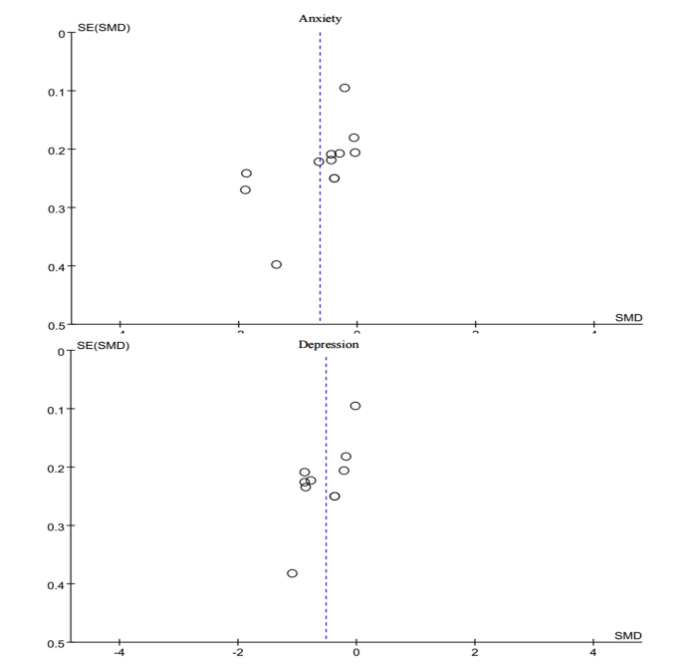
Funnel plots of anxiety and depression. SMD: standard mean difference.

By generating funnel plots, we assessed the publication bias for anxiety and depression. For anxiety, the funnel plot was asymmetrically distributed on both sides of the null line, suggesting a possible publication bias. For depression, the funnel plot was symmetrically distributed, suggesting a low likelihood of publication bias. For stress, the funnel plot analysis was not performed due to the limited number of studies. (Figure 4)

### Level of Evidence

The assessments of anxiety, depression, and stress are shown in Table 3. For anxiety, the funnel plot was asymmetric on both sides of the null line, so the level of evidence was downgraded by one level due to publication bias. For stress, there was a high risk of selection bias and attrition bias for 1 study, so the level of evidence was downgraded by one level for a serious risk of bias. For anxiety, depression, and stress, the heterogeneity was relatively high (*I*^2^>50%) and the 95% CIs were wide, so the level of evidence was reduced by 2 levels due to serious inconsistency and imprecision. Consequently, the level of evidence for depression was low, and for anxiety and stress, it was very low. That said, we have low levels of evidence that the web-based mindfulness-based intervention reduced depressive symptoms in frontline health care workers during the COVID-19 pandemic, yet only very low levels of evidence that it reduced anxiety and stress.

**Table 3:** GRADE (Grading of Recommendations Assessment Development and Evaluation) evidence profiles for outcomes among the trials included in the systematic review.

	Studies, n	Design	Risk of bias	Inconsistency	Indirectness	Imprecision	Other considerations	Mindfulness, n	Control, n	Absolute effect	Quality	Importance
Anxiety (Better indicated by lower values)	7	Randomized trials	No	Serious^a^	No	Serious^b^	Reporting bias^c^	689	704	SMD 0.63 lower (0.96 to 0.31 lower)	+ – – –Very low	Critical
Depression (Better indicated by lower values)	7	Randomized trials	No	Serious^a^	No	Serious^b^	None	587	616	SMD 0.52 lower (0.77 to 0.26 lower)	++ – – Low	Critical
Stress (better indicated by lower values)	3	Randomized trials	Serious^d^	Serious^a^	No	Serious^b^	None	360	314	SMD 0.20 lower (0.35 to 0.05 lower)	+ – – – Very low	Critical

^a^The heterogeneity was high (*I*^2^>50%).

^b^The 95% CIs were wide.

^c^The funnel plot was asymmetrically distributed on both sides of the null line.

^d^There was a high risk of selection bias and attrition bias in 1 study.

## Discussion

### Principal Results

This study aimed to investigate the impact of web-based mindfulness-based therapy on the symptoms of common mental disorders among frontline health care workers during the COVID-19 pandemic. After a comprehensive search, 10 randomized controlled studies that met the inclusion criteria were included. We found that web-based mindfulness-based intervention may promote a reduction in the symptoms of anxiety, depression, and stress among frontline health care workers. This suggests that even during the COVID-19 pandemic, in an environment of fear and depression, web-based mindfulness-based strategies may provide some reduction in the symptoms of anxiety, depression, and stress in some circumstances.

The latest data released by the World Health Organization show that as of June 14, 2023, as many as 767.9 million patients have been diagnosed with COVID-19 worldwide, with 6.94 million cumulative deaths and 286,078 new cases in the last 7 days [42]. The global form of the COVID-19 pandemic remains severe. Health care workers on the front lines of the pandemic are under immense psychological stress and are subject to many common mental disorders, such as post-traumatic stress disorder, anxiety, and depression [43-45]. Moreover, this phenomenon is more evident in nurses than in other medical staff members [5,6]. Health care workers are the most important bulwark in the fight against COVID-19, and they have worked hard to prevent the spread of the pandemic and save the lives of patients. Undoubtedly, their physical and mental health is an important guarantee of victory in the fight against the pandemic. Therefore, while fighting the COVID-19 pandemic, it is crucial to find appropriate intervention strategies to reduce the symptoms of common mental disorders among health care workers.

Mindfulness-based therapy is a psychological intervention that addresses stress, pain, and illness through mindfulness meditation. During the mindfulness-based practice, the participant’s attention is primarily focused on the breath, emotions, thoughts, and body sensations. The practice of mindfulness is generally initiated by focusing on the breath. During the breathing process, an effort is made to feel the state of being in that moment and to focus attention on that state. Focusing on the breath is the foundation of mindfulness-based practice, which promotes self-control, increases personal awareness, and reduces the effects of stress factors [34]. The mechanism by which mindfulness-based interventions reduce the symptoms of common mental disorders in individuals may lie in enhanced signals from brain regions (eg, frontal cortex and hippocampus) involved in emotion regulation in patients who receive mindfulness-based training [46,47]. Mindfulness-based intervention is acceptable and suitable for different age groups, such as older individuals [48], young adults [49,50], and children [51]. It is also suitable for different social groups, such as students [52], pregnant women [53], patients with cancer [54], and health care workers [55,56]. Given the recommendations about maintaining social distance and the overloaded work schedule of health care workers during the COVID-19 pandemic, attending face-to-face psychological counseling is very inconvenient for them. Therefore, tele-counseling is a better option in this situation. Compared to face-to-face interventions, web-based interventions can reduce the risk of COVID-19 infection for health care workers, saving them valuable time so that they can better focus on patient assistance. Although previous studies have shown the effectiveness of web-based mindfulness-based interventions in managing mental disorders among health care workers [14,21,22], there was no global outbreak of COVID-19 at the time, and the daily stress faced by health care workers was likely to be relatively low. In the context of the COVID-19 pandemic, can web-based mindfulness-based interventions still play a beneficial role for health care professionals? Our findings suggest that web-based mindfulness-based interventions could continue to have positive effects on the management of common mental disorders among health care workers, even during the COVID-19 pandemic. This may help reduce the symptoms of mental disorders among frontline health care workers in the fight against such outbreaks.

It is important to note that the pooled heterogeneity of anxiety, depression, and stress is high. Using subgroup analysis, we determined that this heterogeneity stems mainly from the duration of intervention and the types of scales. For example, the study by Ghazanfarpour et al [33] used 2 scales (HADS and SHAI) to assess participants’ anxiety; however, the results of the HADS showed that web-based mindfulness-based interventions reduced anxiety levels in health care workers, but the SHAI did not. In addition, Santamaría-Peláez et al [36] showed that when the web-based mindfulness-based interventions lasted up to 4 weeks, there were no significant differences in anxiety and depression levels between the experimental and control groups; however, when the interventions were conducted for up to 8 weeks, the differences between the groups became significant. In addition, we noted that the meta-analyses had wide CIs, indicating poor precision. Therefore, the results should be interpreted with caution.

Furthermore, we noted that the study by Santamaría-Peláez et al [36] had a 3-month follow-up for participants, and the results of the follow-up were the same as those at the end of the intervention. For example, at 4 weeks, the difference between groups was not significant, nor was the difference at follow-up; at 8 weeks, the difference between groups was significant, as was the difference at follow-up. This suggests that the web-based mindfulness-based interventions may have a long-term effect on participants’ mental regulation. However, there are very few follow-up studies about the effects of web-based mindfulness-based interventions on health care workers’ mental disorders, suggesting that future researchers should focus on the follow-up effects of mindfulness-based interventions.

The strength of this study is that it is the first systematic review and meta-analysis of the impact of web-based mindfulness-based interventions on anxiety, depression, and stress among frontline health care workers during the COVID-19 pandemic. We focused not only on the immediate effects after the web-based mindfulness-based intervention but also on the follow-up effects after the intervention to explore the long-term effects of the web-based mindfulness-based intervention.

### Limitations

This study has several limitations. First, the use of different scales to assess anxiety, depression, and stress in different countries is a major source of heterogeneity. Due to the high heterogeneity and wide CI of the results in the meta-analyses, we should be cautious about the interpretation of the results. Second, possibly owing to the limitations of the pandemic, some studies had short intervention durations, ranging from a single session to 8 sessions, which leaves a lack of evidence for the long-term effects of web-based mindfulness-based interventions on their efficacy in reducing the symptoms of common mental disorders among health care workers. Third, we analyzed both postintervention and follow-up data, which may bring about some bias.

### Future Directions

Given the current trend of the pandemic, regular epidemic prevention and control are the main focus areas. This also suggests that the majority of health care workers need to be prepared to fight the pandemic for a long time. Therefore, safeguarding the mental health of health care workers has long been an exceptionally important matter. It is valuable to develop an easy-to-use app for health care workers to relieve stress and experience relaxation at work. In addition, researchers should make a transition from the short-term to the long-term effects of web-based mindfulness-based therapy on reducing symptoms of common mental disorders in frontline health care workers, with appropriate attention to follow-up effects after the end of the intervention.

### Conclusions

This study systematically reviews the effectiveness of web-based mindfulness-based interventions in the management of common mental disorders among frontline health care workers in the fight against the COVID-19 pandemic. The results showed that web-based mindfulness-based psychotherapy may be effective in reducing the symptoms of anxiety, depression, and stress among health care workers, even during the COVID-19 pandemic, but benefits are likely to be mild on average, and more research is needed before a clear recommendation can be made. In the future, randomized controlled studies with high quality and long intervention times are required to further justify this conclusion.

## Appendix

Multimedia Appendix 1 The search strategy of different databases.

Multimedia Appendix 2 The list of excluded studies and the reasons for their exclusion (18 studies).

Multimedia Appendix 3 Mindfulness-based intervention protocol of each study.

Multimedia Appendix 4 Subgroup analyses for anxiety and depression were performed according to the duration of intervention.

Multimedia Appendix 5 Subgroup analyses for anxiety and depression were performed according to the types of scales.

## Abbreviations

CNKI: China National Knowledge Infrastructure
GRADE: Grading of Recommendations Assessment Development and Evaluation
HADS: Hospital Anxiety and Depression Scale
MBCT: mindfulness-based cognitive therapy
MBSR: mindfulness-based stress reduction
PEDro: Physiotherapy Evidence Database
PICOS: population, intervention, comparison, outcomes, and study design
PRISMA: Preferred Reporting Items for Systematic Reviews and Meta-Analyses
SAS: Self-rating Anxiety Scale
SDS: Self-rating Depression Scale
SHAI: Short Health Anxiety Inventory
SMD: standard mean difference

## Data Availability

Published data used for the systematic review with meta-analysis are available from the authors.
