# Supplementary material for: Effects of Web-Based Mindfulness-Based Interventions on Anxiety, Depression, and Stress Among Frontline Health Care Workers During the COVID-19 Pandemic: Systematic Review and Meta-Analysis
Source: J Med Internet Res. 2023 Aug 29;25:e44000. doi: 10.2196/44000 (PMC10467633; doi:10.2196/44000)
Supplement: Multimedia Appendix 1 [file jmir_v25i1e44000_app1.docx]

**Multimedia Appendix 1.** The search strategy of different databases.

| **PubMed** | **Search terms for query** |
| --- | --- |
| #1 | mindfulness OR mindfulness-based stress reduction OR mindfulness-based cognitive therapy OR mindfulness meditation |
| #2 | COVID-19 OR coronavirus disease 2019 OR SARS-nCoV-2 OR SARS-CoV-2 OR 2019-nCoV OR novel coronavirus OR coronavirus |
| #3 | health care worker OR medical worker OR medical staff OR medical personnel OR nurse OR doctor OR physician |
| #4 | anxiety OR angst OR social anxiety OR anxiousness |
| #5 | depression OR depressive disorder OR emotional depression OR depressive syndrome |
| #6 | stress |
| #7 | randomized controlled OR RCT OR controlled trial OR clinical trial |
| #8 | #4 OR #5 OR #6 |
| #9 | #1 AND #2 AND #3 AND #7 AND #8 |
| **Web of Science** | **Search terms for query** |
| #1 | “mindfulness” OR “mindfulness-based stress reduction” OR “mindfulness-based cognitive therapy” OR “mindfulness meditation” |
| #2 | “health care worker” OR “medical worker” OR “medical staff” OR “medical personnel” OR “nurse” OR “doctor” OR “physician” |
| #3 | “anxiety” OR “angst” OR “social anxiety” OR “anxiousness” OR “depression” OR “depressive disorder” OR “emotional depression” OR “depressive syndrome” OR “stress” |
| #4 | “randomized controlled” OR “RCT” OR “controlled trial” OR “clinical trial” |
| #5 | #1 AND #2 AND #3 AND #4 |
| Filters | from 2020 - 2022 |
| **Embase** | **Search terms for query** |
| #1 | mindfulness:ab,ti,kw OR mindfulness-based stress reduction:ab,ti,kw OR mindfulness-based cognitive therapy:ab,ti,kw OR mindfulness meditation:ab,ti,kw |
| #2 | health care worker:ab,ti,kw OR medical worker:ab,ti,kw OR medical staff:ab,ti,kw OR medical personnel:ab,ti,kw OR nurse:ab,ti,kw OR doctor:ab,ti,kw OR physician:ab,ti,kw |
| #3 | anxiety:ab,ti,kw OR angst:ab,ti,kw OR social anxiety:ab,ti,kw OR anxiousness:ab,ti,kw |
| #4 | depression:ab,ti,kw OR depressive disorder:ab,ti,kw OR emotional depression:ab,ti,kw OR depressive syndrome:ab,ti,kw |
| #5 | stress:ab,ti,kw |
| #6 | randomized controlled:ab,ti,kw OR RCT:ab,ti,kw OR controlled trial:ab,ti,kw OR clinical trial:ab,ti,kw |
| #7 | #4 OR #5 OR #6 |
| #8 | #1 AND #2 AND #3 AND #7 AND #8 |
| Filters | from 2020 - 2022 |
| **Cochrane** | **Search terms for query** |
| #1 | mindfulness:ab,ti,kw OR mindfulness meditation:ab,ti,kw |
| #2 | health care worker:ab,ti,kw OR medical worker:ab,ti,kw OR medical staff:ab,ti,kw OR medical personnel:ab,ti,kw OR nurse:ab,ti,kw OR doctor:ab,ti,kw OR physician:ab,ti,kw |
| #3 | anxiety:ab,ti,kw OR angst:ab,ti,kw OR social anxiety:ab,ti,kw OR anxiousness:ab,ti,kw |
| #4 | depression:ab,ti,kw OR depressive disorder:ab,ti,kw OR emotional depression:ab,ti,kw OR depressive syndrome:ab,ti,kw |
| #5 | stress:ab,ti,kw |
| #6 | #3 OR #4 OR #5 |
| #7 | #1 AND #2 AND #3 AND #6 |
| Filters | from 2020 - 2022 |
| **Scopus** | **Search terms for query** |
| #1 | “mindfulness” OR “mindfulness-based stress reduction” OR “mindfulness-based cognitive therapy” OR “mindfulness meditation” |
| #2 | “COVID-19” OR “coronavirus disease 2019” OR “SARS-nCoV-2” OR “SARS-CoV-2” OR “2019-nCoV” OR “novel coronavirus” OR “coronavirus” |
| #3 | “health care worker” OR “medical worker” OR “medical staff” OR “medical personnel” OR “nurse” OR “doctor” OR “physician” |
| #4 | “anxiety” OR “angst” OR “social anxiety” OR “anxiousness” OR “depression” OR “depressive disorder” OR “emotional depression” OR “depressive syndrome” OR “stress” |
| #5 | “randomized controlled” OR “RCT” OR “controlled trial” OR “clinical trial” |
| #6 | #1 AND #2 AND #3 AND #4 AND #5 |
| **ScienceDirect** | **Search terms for query** |
| #1 | mindfulness AND COVID-19 AND (health care worker OR nurse OR doctor) AND (anxiety OR depression OR stress) AND (randomized controlled) |
| **SinoMed** | **Search terms for query** |
| #1 | 正念 OR 正念减压疗法 OR 正念认知疗法 OR 正念冥想 |
| #2 | 抑郁 OR 焦虑 OR 情志异常 OR 抑郁症状 OR 抑郁情绪 OR 压力 |
| #3 | 病毒性肺炎 OR COVID-19 OR 新冠肺炎 OR 新型冠状病毒肺炎 |
| #4 | 护士 OR 医护人员 OR 医生 |
| #5 | 随机对照 OR 随机分组 OR 随机 OR RCT |
| #6 | #1 AND #2 AND #3 AND #4 AND #5 |
| **CNKI** | **Search terms for query** |
| #1 | （正念 OR 正念减压疗法 OR 正念认知疗法 OR 正念冥想）AND （抑郁 OR 焦虑 OR 情志异常 OR 抑郁症状 OR 抑郁情绪 OR 压力）AND（病毒性肺炎 OR COVID-19 OR 新冠肺炎 OR 新型冠状病毒肺炎）AND（护士 OR 医护人员 OR 医生） |
| **WanFang** | **Search terms for query** |
| #1 | 正念 OR 正念减压疗法 OR 正念认知疗法 OR 正念冥想 |
| #2 | 抑郁 OR 焦虑 OR 情志异常 OR 抑郁症状 OR 抑郁情绪 OR 压力 |
| #3 | 病毒性肺炎 OR COVID-19 OR 新冠肺炎 OR 新型冠状病毒肺炎 |
| #4 | 护士 OR 医护人员 OR 医生 |
| #5 | 随机对照 OR 随机分组 OR 随机 OR RCT |
| #6 | #1 AND #2 AND #3 AND #4 AND #5 |
