# Supplementary material for: Effects of Web-Based Mindfulness-Based Interventions on Anxiety, Depression, and Stress Among Frontline Health Care Workers During the COVID-19 Pandemic: Systematic Review and Meta-Analysis
Source: J Med Internet Res. 2023 Aug 29;25:e44000. doi: 10.2196/44000 (PMC10467633; doi:10.2196/44000)
Supplement: Multimedia Appendix 2 [file jmir_v25i1e44000_app2.docx]

**Multimedia Appendix 2.** The list of excluded studies and the reasons for their exclusion (18 studies).

| **Study** | **Title** | **Reason** |
| --- | --- | --- |
| DeTore et al. 2022 | Promoting resilience in healthcare workers during the COVID-19 pandemic with a brief online intervention | Not randomized controlled trial |
| Dincer et al. 2021 | The effect of Emotional Freedom Techniques on nurses’ stress, anxiety, and burnout levels during the COVID-19 pandemic: A randomized controlled trial | Not mindfulness-based intervention |
| Errazuriz et al. 2022 | Effects of mindfulness-based stress reduction on psychological distress in health workers: a three-arm parallel randomized controlled trial | The timing of the intervention preceded the COVID-19 outbreak |
| Ghawadra et al. 2020 | The effect of mindfulness-based training on stress, anxiety, depression and job satisfaction among ward nurses: a randomized control trial | The timing of the intervention preceded the COVID-19 outbreak |
| Gomutbutra et al. 2022 | Effect of intensive weekend mindfulness-based intervention on BDNF, mitochondria function, and anxiety. A randomized, crossover clinical trial | The participants were not only medical staff, but also students |
| Li et al. 2020 | 正念减压法在新型冠状病毒肺炎疫情防控一线护士中的应用[The application of mindfulness-based stress reduction in frontline nurses in COVID-19 outbreak prevention and control] | Not web-based intervention |
| Mealer et al. 2021 | A Mindfulness-Based Cognitive Therapy (MBCT) Intervention to Improve Resilience and Mitigate Symptoms of Burnout Syndrome in Critical Care Nurses: Results of a Randomized Trial | The timing of the intervention preceded the COVID-19 outbreak |
| Nourian et al. 2021 | The Impact of an Online Mindfulness-Based Stress Reduction Program on Sleep Quality of Nurses Working in COVID-19 Care Units: A Clinical Trial | The outcome indicators do not meet the inclusion criteria |
| Pérez et al. 2022 | Mindfulness-Based Intervention for the Reduction of Compassion Fatigue and Burnout in Nurse Caregivers of Institutionalized Older Persons with Dementia: A Randomized Controlled Trial | The outcome indicators do not meet the inclusion criteria |
| Rodriguez-Vega et al. 2020 | Implementation of a Mindfulness-Based Crisis Intervention for Frontline Healthcare Workers During the COVID-19 Outbreak in a Public General Hospital in Madrid, Spain | Not randomized controlled trial |
| Seidel et al. 2021 | Brief Mindfulness Practice Course for Healthcare Providers | The outcome indicators do not meet the inclusion criteria |
| Serrano-Ripoll et al. 2021 | Effect of a mobile-based intervention on mental health in frontline healthcare workers against COVID-19: Protocol for a randomized controlled trial | Protocol |
| Strauss et al. 2021 | Reducing stress and promoting well-being in healthcare workers using mindfulness-based cognitive therapy for life | The timing of the intervention preceded the COVID-19 outbreak |
| Taylor et al. 2022 | Health Care Workers' Need for Headspace: findings From a Multisite Definitive Randomized Controlled Trial of an Unguided Digital Mindfulness-Based Self-help App to Reduce Healthcare Worker Stress | The timing of the intervention preceded the COVID-19 outbreak |
| Thimmapuram et al. 2021 | Heartfulness meditation improves loneliness and sleep in physicians and advance practice providers during COVID-19 pandemic | The outcome indicators do not meet the inclusion criteria |
| Xu et al. 2021 | A mobile mindfulness intervention for emergency department staff to improve stress and wellbeing: A qualitative study | Not randomized controlled trial |
| Ye et al. 2020 | 新型冠状病毒肺炎形势下正念减压疗法对医务人员的心理干预研究[Psychological intervention of MBSR on medical staff under COVID-19 situation] | Conference abstract |
| Zeller et al. 2021 | Mindfulness Training to Improve Nurse Clinical Performance: a Pilot Study | Not web-based intervention |
