# Supplementary material for: Effects of Web-Based Mindfulness-Based Interventions on Anxiety, Depression, and Stress Among Frontline Health Care Workers During the COVID-19 Pandemic: Systematic Review and Meta-Analysis
Source: J Med Internet Res. 2023 Aug 29;25:e44000. doi: 10.2196/44000 (PMC10467633; doi:10.2196/44000)
Supplement: Multimedia Appendix 3 [file jmir_v25i1e44000_app3.docx]

**Multimedia Appendix 3.** Mindfulness-based intervention protocol of each study.

| **Study** | **Experimental group** | **Control group** | **Intervention time** |
| --- | --- | --- | --- |
| Fiol-DeRoque et al. 2021 | Intervention based on cognitive-behavioral therapy and mindfulness approaches, included written and audiovisual content targeting four areas: emotional skills, healthy lifestyle behavior, work stress and burnout, and social support. | Participants were briefed on written information about mental health care for health care workers during the COVID-19 pandemic. | 2 weeks |
| Ghazanfarpour et al. 2021 | Counseling was implemented through voice or video calls, text chats, and video clips shared on WhatsApp, in seven sessions on seven consecutive days Depending on the number of questions and participants’ experience, the duration of audio and video exchange in each session varied from a minimum of 45 minutes up to 90 minutes. | No intervention. | 7 sessions |
| Li et al. 2020 | Participants practiced brief mindfulness meditation for 15 minutes daily for 16 days. | No intervention. | 16 days |
| Santamaría-Peláez et al. 2021 | Experimental group 1: Participants belonged to an abbreviated training program, which consisted of 4 weekly sessions lasting 150 min and 15 min of daily practices at home.  Experimental group 2: A standard mindfulness and self-compassion program, consisting of 8 (one per week) sessions of 150 min with the daily practice for 30 min at home. | No intervention. | 4 weeks and 8 weeks |
| Yıldırım et al. 2022 | During the session, participants were asked to relax in a setting as quiet and calm as possible. Mindfulness-based breathing and music therapy was applied to each subgroup of nurses for approximately 30 minutes in a single session. | Participants were asked to relax in a quiet and calm setting for the next 30 minutes. | 1 session |
| Zeng et al. 2022 | The participants intervened with mindfulness-based stress reduction, set up information exchange platforms such as WeChat group and Official Account, and made intervention plans and related courseware according to the questionnaire on the psychological status of medical staff during COVID-19. | Participants received psychological intervention based on WeChat group, and shared and discussed the information related to the promotion of mental health in the group. | 4 weeks |
| Huang et al. 2021 | Using the practice-based approach, the consultant led the subjects to carry out practical training, training one topic a week, each topic for 5 days, and practicing 40 min every day, including 30 min training and 10 min interactive sharing. | Make mental health education videos, hold 40 min health lectures every night, including 30 min lectures, 10 min interactive sharing, and distribute crisis science manuals for front-line health care workers to read. | 8 weeks |
| Tong et al. 2021 | The observation group received online mindfulness intervention 4 times in 4 weeks on the basis of general psychological intervention, and the intervention time ranged from 65 minutes to 94 minutes. | The control group was treated with general psychological intervention for 4 weeks, once a week for 50 minutes each time. | 4 weeks |
| Wang et al. 2020 | The study group received 4-week mindfulness-based stress reduction intervention led by mindfulness therapists on the basis of the control group. Make online mindfulness decompression courseware and intervene with the help of Official Account, WeChat and DingTalk platforms. | Routine supportive psychological counseling: guide participants to objectively and rationally understand the epidemic situation, provide necessary protective materials and living security, pay more attention to encouragement, timely rest and recuperation, and avoid fatigue. | 4 weeks |
| Zou et al. 2021 | Participants received mindfulness meditation combined with positive psychological intervention, including basic mindfulness, mindfulness relaxation, mindfulness training, mindfulness meditation, finding advantages, learning gratitude, practicing positive language and feeling a good life. | The control group did not give any treatment. | 8 weeks |
