# Supplementary figures and images for: Effects of Web-Based Mindfulness-Based Interventions on Anxiety, Depression, and Stress Among Frontline Health Care Workers During the COVID-19 Pandemic: Systematic Review and Meta-Analysis
Source: J Med Internet Res. 2023 Aug 29;25:e44000. doi: 10.2196/44000 (PMC10467633; doi:10.2196/44000)

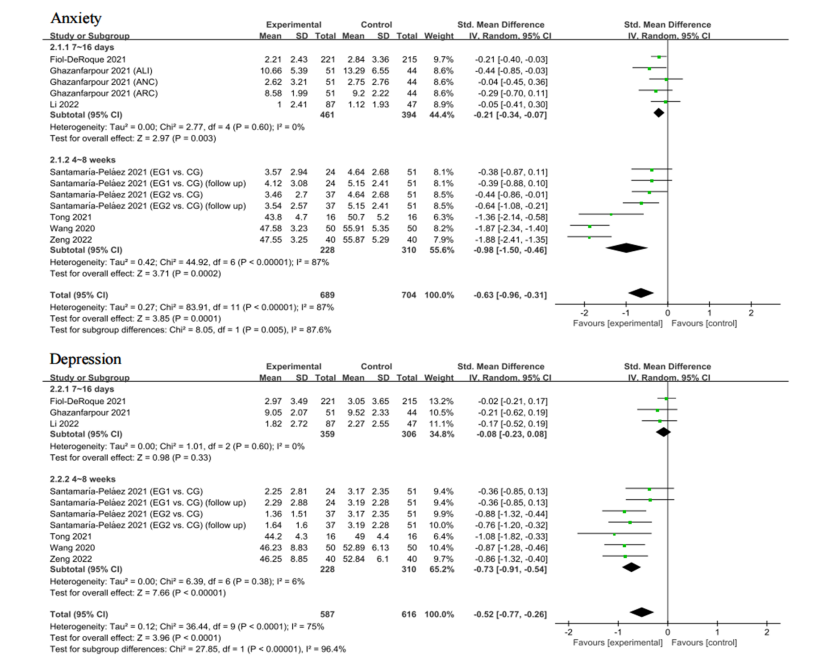

Supplement: Multimedia Appendix 4 [file jmir_v25i1e44000_app4.png]

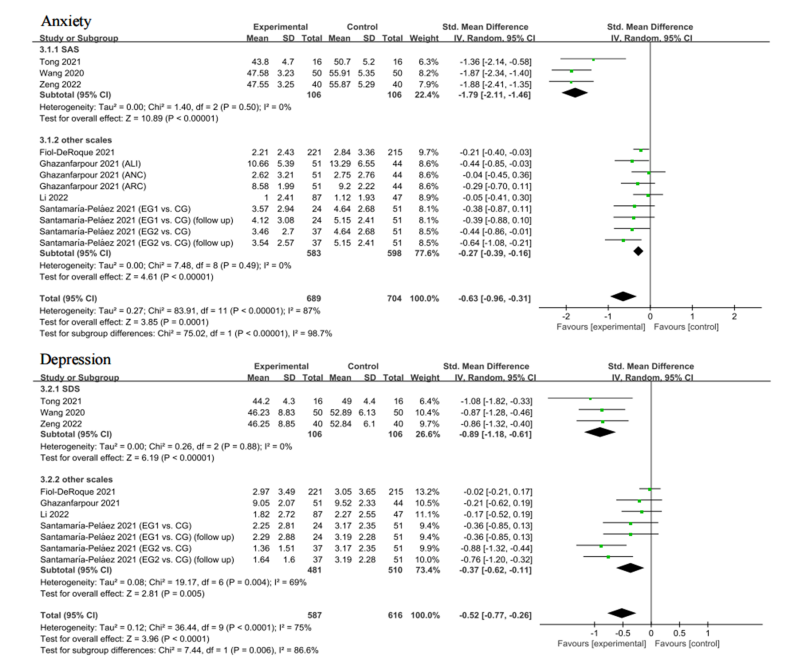

Supplement: Multimedia Appendix 5 [file jmir_v25i1e44000_app5.png]
